# Author Correction: Genotype-stratified treatment for monogenic insulin resistance: a systematic review

**DOI:** 10.1038/s43856-024-00482-2

**Published:** 2024-03-26

**Authors:** Robert K. Semple, Kashyap A. Patel, Sungyoung Auh, ADA/EASD PMDI, Deirdre K. Tobias, Jordi Merino, Abrar Ahmad, Catherine Aiken, Jamie L. Benham, Dhanasekaran Bodhini, Amy L. Clark, Kevin Colclough, Rosa Corcoy, Sara J. Cromer, Daisy Duan, Jamie L. Felton, Ellen C. Francis, Pieter Gillard, Véronique Gingras, Romy Gaillard, Eram Haider, Alice Hughes, Jennifer M. Ikle, Laura M. Jacobsen, Anna R. Kahkoska, Jarno L. T. Kettunen, Raymond J. Kreienkamp, Lee-Ling Lim, Jonna M. E. Männistö, Robert Massey, Niamh-Maire Mclennan, Rachel G. Miller, Mario Luca Morieri, Jasper Most, Rochelle N. Naylor, Bige Ozkan, Kashyap Amratlal Patel, Scott J. Pilla, Katsiaryna Prystupa, Sridaran Raghaven, Mary R. Rooney, Martin Schön, Zhila Semnani-Azad, Magdalena Sevilla-Gonzalez, Pernille Svalastoga, Wubet Worku Takele, Claudia Ha-ting Tam, Anne Cathrine B. Thuesen, Mustafa Tosur, Amelia S. Wallace, Caroline C. Wang, Jessie J. Wong, Jennifer M. Yamamoto, Katherine Young, Chloé Amouyal, Mette K. Andersen, Maxine P. Bonham, Mingling Chen, Feifei Cheng, Tinashe Chikowore, Sian C. Chivers, Christoffer Clemmensen, Dana Dabelea, Adem Y. Dawed, Aaron J. Deutsch, Laura T. Dickens, Linda A. DiMeglio, Monika Dudenhöffer-Pfeifer, Carmella Evans-Molina, María Mercè Fernández-Balsells, Hugo Fitipaldi, Stephanie L. Fitzpatrick, Stephen E. Gitelman, Mark O. Goodarzi, Jessica A. Grieger, Marta Guasch-Ferré, Nahal Habibi, Torben Hansen, Chuiguo Huang, Arianna Harris-Kawano, Heba M. Ismail, Benjamin Hoag, Randi K. Johnson, Angus G. Jones, Robert W. Koivula, Aaron Leong, Gloria K. W. Leung, Ingrid M. Libman, Kai Liu, S. Alice Long, William L. Lowe, Robert W. Morton, Ayesha A. Motala, Suna Onengut-Gumuscu, James S. Pankow, Maleesa Pathirana, Sofia Pazmino, Dianna Perez, John R. Petrie, Camille E. Powe, Alejandra Quinteros, Rashmi Jain, Debashree Ray, Mathias Ried-Larsen, Zeb Saeed, Vanessa Santhakumar, Sarah Kanbour, Sudipa Sarkar, Gabriela S. F. Monaco, Denise M. Scholtens, Elizabeth Selvin, Wayne Huey-Herng Sheu, Cate Speake, Maggie A. Stanislawski, Nele Steenackers, Andrea K. Steck, Norbert Stefan, Julie Støy, Rachael Taylor, Sok Cin Tye, Gebresilasea Gendisha Ukke, Marzhan Urazbayeva, Bart Van der Schueren, Camille Vatier, John M. Wentworth, Wesley Hannah, Sara L. White, Gechang Yu, Yingchai Zhang, Shao J. Zhou, Jacques Beltrand, Michel Polak, Ingvild Aukrust, Elisa de Franco, Sarah E. Flanagan, Kristin A. Maloney, Andrew McGovern, Janne Molnes, Mariam Nakabuye, Pål Rasmus Njølstad, Hugo Pomares-Millan, Michele Provenzano, Cécile Saint-Martin, Cuilin Zhang, Yeyi Zhu, Sungyoung Auh, Russell de Souza, Andrea J. Fawcett, Chandra Gruber, Eskedar Getie Mekonnen, Emily Mixter, Diana Sherifali, Robert H. Eckel, John J. Nolan, Louis H. Philipson, Rebecca J. Brown, Liana K. Billings, Kristen Boyle, Tina Costacou, John M. Dennis, Jose C. Florez, Anna L. Gloyn, Maria F. Gomez, Peter A. Gottlieb, Siri Atma W. Greeley, Kurt Griffin, Andrew T. Hattersley, Irl B. Hirsch, Marie-France Hivert, Korey K. Hood, Jami L. Josefson, Soo Heon Kwak, Lori M. Laffel, Siew S. Lim, Ruth J. F. Loos, Ronald C. W. Ma, Chantal Mathieu, Nestoras Mathioudakis, James B. Meigs, Shivani Misra, Viswanathan Mohan, Rinki Murphy, Richard Oram, Katharine R. Owen, Susan E. Ozanne, Ewan R. Pearson, Wei Perng, Toni I. Pollin, Rodica Pop-Busui, Richard E. Pratley, Leanne M. Redman, Maria J. Redondo, Rebecca M. Reynolds, Jennifer L. Sherr, Emily K. Sims, Arianne Sweeting, Tiinamaija Tuomi, Miriam S. Udler, Kimberly K. Vesco, Tina Vilsbøll, Robert Wagner, Stephen S. Rich, Paul W. Franks, Rebecca J. Brown

**Affiliations:** 1 Centre for Cardiovascular Science, Queen’s Medical Research Institute, University of Edinburgh, Edinburgh, UK; 2 MRC Human Genetics Unit, Institute of Genetics and Cancer, University of Edinburgh, Edinburgh, UK; 3 Department of Clinical and Biomedical Sciences, University of Exeter Medical School, Exeter, UK; 4 Department of Diabetes and Endocrinology, Royal Devon and Exeter NHS Foundation Trust, Exeter, UK; 5 National Institute of Diabetes and Digestive and Kidney Diseases, National Institutes of Health, Bethesda, MD USA; 6 Division of Preventative Medicine, Department of Medicine, Brigham and Women’s Hospital and Harvard Medical School, Boston, MA USA; 7 Department of Nutrition, Harvard T.H. Chan School of Public Health, Boston, MA USA; 8 Novo Nordisk Foundation Center for Basic Metabolic Research, Faculty of Health and Medical Sciences, University of Copenhagen, Copenhagen, Denmark; 9 Diabetes Unit, Endocrine Division, Massachusetts General Hospital, Boston, MA USA; 10 Center for Genomic Medicine, Massachusetts General Hospital, Boston, MA USA; 11 Department of Clinical Sciences, Lund University Diabetes Centre, Lund University Malmö, Sweden; 12 Department of Obstetrics and Gynaecology, the Rosie Hospital, Cambridge, UK; 13 NIHR Cambridge Biomedical Research Centre, University of Cambridge, Cambridge, UK; 14 Departments of Medicine and Community Health Sciences, Cumming School of Medicine, University of Calgary, Calgary, AB Canada; 15 Department of Molecular Genetics, Madras Diabetes Research Foundation, Chennai, India; 16 Division of Pediatric Endocrinology, Department of Pediatrics, Saint Louis University School of Medicine, SSM Health Cardinal Glennon Children’s Hospital, St. Louis, MO USA; 17 CIBER-BBN, ISCIII, Madrid, Spain; 18 Institut d’Investigació Biomèdica Sant Pau (IIB SANT PAU), Barcelona, Spain; 19 Departament de Medicina, Universitat Autònoma de Barcelona, Bellaterra, Spain; 20 Programs in Metabolism and Medical & Population Genetics, Broad Institute, Cambridge, MA USA; 21 Department of Medicine, Harvard Medical School, Boston, MA USA; 22 Division of Endocrinology, Diabetes and Metabolism, Johns Hopkins University School of Medicine, Baltimore, MD USA; 23 Department of Pediatrics, Indiana University School of Medicine, Indianapolis, IN USA; 24 Herman B Wells Center for Pediatric Research, Indiana University School of Medicine, Indianapolis, IN USA; 25 Center for Diabetes and Metabolic Diseases, Indiana University School of Medicine, Indianapolis, IN USA; 26 Department of Biostatistics and Epidemiology, Rutgers School of Public Health, Piscataway, NJ USA; 27 University Hospital Leuven, Leuven, Belgium; 28 Department of Nutrition, Université de Montréal, Montreal, QC Canada; 29 Research Center, Sainte-Justine University Hospital Center, Montreal, QC Canada; 30 Department of Pediatrics, Erasmus Medical Center, Rotterdam, The Netherlands; 31 Division of Population Health & Genomics, School of Medicine, University of Dundee, Dundee, UK; 32 Department of Pediatrics, Stanford School of Medicine, Stanford University, Stanford, CA USA; 33 Stanford Diabetes Research Center, Stanford School of Medicine, Stanford University, Stanford, CA USA; 34 University of Florida, Gainesville, FL USA; 35 Department of Nutrition, University of North Carolina at Chapel Hill, Chapel Hill, NC USA; 36 Helsinki University Hospital, Abdominal Centre/Endocrinology, Helsinki, Finland; 37 Folkhalsan Research Center, Helsinki, Finland; 38 Institute for Molecular Medicine Finland FIMM, University of Helsinki, Helsinki, Finland; 39 Department of Pediatrics, Division of Endocrinology, Boston Children’s Hospital, Boston, MA USA; 40 Department of Medicine, Faculty of Medicine, University of Malaya, Kuala Lumpur, Malaysia; 41 Asia Diabetes Foundation, Hong Kong SAR, China; 42 Department of Medicine & Therapeutics, Chinese University of Hong Kong, Hong Kong SAR, China; 43 Departments of Pediatrics and Clinical Genetics, Kuopio University Hospital, Kuopio, Finland; 44 Department of Medicine, University of Eastern Finland, Kuopio, Finland; 45 Department of Epidemiology, University of Pittsburgh, Pittsburgh, PA USA; 46 Metabolic Disease Unit, University Hospital of Padova, Padova, Italy; 47 Department of Medicine, University of Padova, Padova, Italy; 48 Department of Orthopedics, Zuyderland Medical Center, Sittard-Geleen, The Netherlands; 49 Departments of Pediatrics and Medicine, University of Chicago, Chicago, IL USA; 50 Welch Center for Prevention, Epidemiology, and Clinical Research, Johns Hopkins Bloomberg School of Public Health, Baltimore, MD USA; 51 Ciccarone Center for the Prevention of Cardiovascular Disease, Johns Hopkins School of Medicine, Baltimore, MD USA; 52 Department of Medicine, Johns Hopkins University, Baltimore, MD USA; 53 Department of Health Policy and Management, Johns Hopkins University Bloomberg School of Public Health, Baltimore, MD USA; 54 Institute for Clinical Diabetology, German Diabetes Center, Leibniz Center for Diabetes Research at Heinrich Heine University Düsseldorf, Auf’m Hennekamp 65, 40225 Düsseldorf, Germany; 55 German Center for Diabetes Research (DZD), Ingolstädter Landstraße 1, 85764 Neuherberg, Germany; 56 Section of Academic Primary Care, US Department of Veterans Affairs Eastern Colorado Health Care System, Aurora, CO USA; 57 Department of Medicine, University of Colorado School of Medicine, Aurora, CO USA; 58 Department of Epidemiology, Johns Hopkins Bloomberg School of Public Health, Baltimore, MD USA; 59 Institute of Experimental Endocrinology, Biomedical Research Center, Slovak Academy of Sciences, Bratislava, Slovakia; 60 Clinical and Translational Epidemiology Unit, Massachusetts General Hospital, Boston, MA USA; 61 Mohn Center for Diabetes Precision Medicine, Department of Clinical Science, University of Bergen, Bergen, Norway; 62 Children and Youth Clinic, Haukeland University Hospital, Bergen, Norway; 63 Eastern Health Clinical School, Monash University, Melbourne, VIC Australia; 64 Laboratory for Molecular Epidemiology in Diabetes, Li Ka Shing Institute of Health Sciences, The Chinese University of Hong Kong, Hong Kong, China; 65 Hong Kong Institute of Diabetes and Obesity, The Chinese University of Hong Kong, Hong Kong, China; 66 Department of Pediatrics, Baylor College of Medicine, Houston, TX USA; 67 Division of Pediatric Diabetes and Endocrinology, Texas Children’s Hospital, Houston, TX USA; 68 Children’s Nutrition Research Center, USDA/ARS, Houston, TX USA; 69 Stanford University School of Medicine, Stanford, CA USA; 70 Internal Medicine, University of Manitoba, Winnipeg, MB Canada; 71 Department of Diabetology, APHP, Paris, France; 72 Sorbonne Université, INSERM, NutriOmic team, Paris, France; 73 Department of Nutrition, Dietetics and Food, Monash University, Melbourne, VIC Australia; 74 Monash Centre for Health Research and Implementation, Monash University, Clayton, VIC Australia; 75 Health Management Center, The Second Affiliated Hospital of Chongqing Medical University, Chongqing Medical University, Chongqing, China; 76 MRC/Wits Developmental Pathways for Health Research Unit, Department of Paediatrics, Faculty of Health Sciences, University of the Witwatersrand, Johannesburg, South Africa; 77 Channing Division of Network Medicine, Brigham and Women’s Hospital, Boston, MA USA; 78 Sydney Brenner Institute for Molecular Bioscience, Faculty of Health Sciences, University of the Witwatersrand, Johannesburg, South Africa; 79 Department of Women and Children’s health, King’s College London, London, UK; 80 Lifecourse Epidemiology of Adiposity and Diabetes (LEAD) Center, University of Colorado Anschutz Medical Campus, Aurora, CO USA; 81 Section of Adult and Pediatric Endocrinology, Diabetes and Metabolism, Kovler Diabetes Center, University of Chicago, Chicago, USA; 82 Department of Pediatrics, Riley Hospital for Children, Indiana University School of Medicine, Indianapolis, IN USA; 83 Richard L. Roudebush VAMC, Indianapolis, IN USA; 84 Biomedical Research Institute Girona, IdIBGi, Girona, Spain; 85 Diabetes, Endocrinology and Nutrition Unit Girona, University Hospital Dr Josep Trueta, Girona, Spain; 86 Institute of Health System Science, Feinstein Institutes for Medical Research, Northwell Health, Manhasset, NY USA; 87 University of California at San Francisco, Department of Pediatrics, Diabetes Center, San Francisco, CA USA; 88 Division of Endocrinology, Diabetes and Metabolism, Cedars-Sinai Medical Center, Los Angeles, CA USA; 89 Department of Medicine, Cedars-Sinai Medical Center, Los Angeles, CA USA; 90 Adelaide Medical School, Faculty of Health and Medical Sciences, The University of Adelaide, Adelaide, Australia; 91 Robinson Research Institute, The University of Adelaide, Adelaide, Australia; 92 Department of Public Health and Novo Nordisk Foundation Center for Basic Metabolic Research, Faculty of Health and Medical Sciences, University of Copenhagen, 1014 Copenhagen, Denmark; 93 Division of Endocrinology and Diabetes, Department of Pediatrics, Sanford Children’s Hospital, Sioux Falls, SD USA; 94 University of South Dakota School of Medicine, E Clark St, Vermillion, SD USA; 95 Department of Biomedical Informatics, University of Colorado Anschutz Medical Campus, Aurora, CO USA; 96 Department of Epidemiology, Colorado School of Public Health, Aurora, CO USA; 97 Royal Devon University Healthcare NHS Foundation Trust, Exeter, UK; 98 Oxford Centre for Diabetes, Endocrinology and Metabolism, University of Oxford, Oxford, UK; 99 Division of General Internal Medicine, Massachusetts General Hospital, Boston, MA USA; 100 UPMC Children’s Hospital of Pittsburgh, Pittsburgh, PA USA; 101 Center for Translational Immunology, Benaroya Research Institute, Seattle, WA USA; 102 Department of Medicine, Northwestern University Feinberg School of Medicine, Chicago, IL USA; 103 Department of Pathology & Molecular Medicine, McMaster University, Hamilton, Canada; 104 Population Health Research Institute, Hamilton, Canada; 105 Department of Translational Medicine, Medical Science, Novo Nordisk Foundation, Tuborg Havnevej 19, 2900 Hellerup, Denmark; 106 Department of Diabetes and Endocrinology, Nelson R Mandela School of Medicine, University of KwaZulu-Natal, Durban, South Africa; 107 Center for Public Health Genomics, Department of Public Health Sciences, University of Virginia, Charlottesville, VA USA; 108 Division of Epidemiology and Community Health, School of Public Health, University of Minnesota, Minneapolis, MN USA; 109 Department of Chronic Diseases and Metabolism, Clinical and Experimental Endocrinology, KU Leuven, Leuven, Belgium; 110 School of Health and Wellbeing, College of Medical, Veterinary and Life Sciences, University of Glasgow, Glasgow, UK; 111 Department of Obstetrics, Gynecology, and Reproductive Biology, Massachusetts General Hospital and Harvard Medical School, Boston, MA USA; 112 Sanford Children’s Specialty Clinic, Sioux Falls, SD USA; 113 Department of Pediatrics, Sanford School of Medicine, University of South Dakota, Sioux Falls, SD USA; 114 Department of Biostatistics, Johns Hopkins Bloomberg School of Public Health, Baltimore, Maryland USA; 115 Centre for Physical Activity Research, Rigshospitalet, Copenhagen, Denmark; 116 Institute for Sports and Clinical Biomechanics, University of Southern Denmark, Odense, Denmark; 117 Department of Medicine, Division of Endocrinology, Diabetes and Metabolism, Indiana University School of Medicine, Indianapolis, IN USA; 118 AMAN Hospital, Doha, Qatar; 119 Department of Preventive Medicine, Division of Biostatistics, Northwestern University Feinberg School of Medicine, Chicago, IL USA; 120 Institute of Molecular and Genomic Medicine, National Health Research Institutes, Taipei City, Taiwan; 121 Divsion of Endocrinology and Metabolism, Taichung Veterans General Hospital, Taichung, Taiwan; 122 Division of Endocrinology and Metabolism, Taipei Veterans General Hospital, Taipei, Taiwan; 123 Center for Interventional Immunology, Benaroya Research Institute, Seattle, WA USA; 124 Barbara Davis Center for Diabetes, University of Colorado Anschutz Medical Campus, Aurora, CO USA; 125 University Hospital of Tübingen, Tübingen, Germany; 126 Institute of Diabetes Research and Metabolic Diseases (IDM), Helmholtz Center Munich, Neuherberg, Germany; 127 Steno Diabetes Center Aarhus, Aarhus University Hospital, Aarhus, Denmark; 128 University of Newcastle, Newcastle upon Tyne, UK; 129 Sections on Genetics and Epidemiology, Joslin Diabetes Center, Harvard Medical School, Boston, MA USA; 130 Department of Clinical Pharmacy and Pharmacology, University Medical Center Groningen, Groningen, The Netherlands; 131 Gastroenterology, Baylor College of Medicine, Houston, TX USA; 132 Department of Endocrinology, University Hospitals Leuven, Leuven, Belgium; 133 Sorbonne University, Inserm U938, Saint-Antoine Research Centre, Institute of Cardiometabolism and Nutrition, Paris, 75012 France; 134 Department of Endocrinology, Diabetology and Reproductive Endocrinology, Assistance Publique-Hôpitaux de Paris, Saint-Antoine University Hospital, National Reference Center for Rare Diseases of Insulin Secretion and Insulin Sensitivity (PRISIS), Paris, France; 135 Royal Melbourne Hospital Department of Diabetes and Endocrinology, Parkville, Vic Australia; 136 Walter and Eliza Hall Institute, Parkville, VIC Australia; 137 University of Melbourne Department of Medicine, Parkville, VIC Australia; 138 Deakin University, Melbourne, Australia; 139 Department of Epidemiology, Madras Diabetes Research Foundation, Chennai, India; 140 Department of Diabetes and Endocrinology, Guy’s and St Thomas’ Hospitals NHS Foundation Trust, London, UK; 141 School of Agriculture, Food and Wine, University of Adelaide, Adelaide, Australia; 142 Institut Cochin, Inserm U 10116 Paris, France; 143 Pediatric endocrinology and diabetes, Hopital Necker Enfants Malades, APHP Centre, université de Paris, Paris, France; 144 Department of Medical Genetics, Haukeland University Hospital, Bergen, Norway; 145 Department of Medicine, University of Maryland School of Medicine, Baltimore, MD USA; 146 Department of Epidemiology, Geisel School of Medicine at Dartmouth, Hanover, NH USA; 147 Nephrology, Dialysis and Renal Transplant Unit, IRCCS—Azienda Ospedaliero-Universitaria di Bologna, Alma Mater Studiorum University of Bologna, Bologna, Italy; 148 Department of Medical Genetics, AP-HP Pitié-Salpêtrière Hospital, Sorbonne University, Paris, France; 149 Global Center for Asian Women’s Health, Yong Loo Lin School of Medicine, National University of Singapore, Singapore, Singapore; 150 Department of Obstetrics and Gynecology, Yong Loo Lin School of Medicine, National University of Singapore, Singapore, Singapore; 151 Kaiser Permanente Northern California Division of Research, Oakland, CA USA; 152 Department of Epidemiology and Biostatistics, University of California San Francisco, California, CA USA; 153 National Institute of Diabetes and Digestive and Kidney Diseases, National Institutes of Health, Bethesda, MD USA; 154 Department of Health Research Methods, Evidence, and Impact, Faculty of Health Sciences, McMaster University, Hamilton, ON Canada; 155 Ann & Robert H. Lurie Children’s Hospital of Chicago, Department of Pediatrics, Northwestern University Feinberg School of Medicine, Chicago, IL USA; 156 Department of Clinical and Organizational Development, Chicago, IL USA; 157 American Diabetes Association, Arlington, VA USA; 158 College of Medicine and Health Sciences, University of Gondar, Gondar, Ethiopia; 159 Global Health Institute, Faculty of Medicine and Health Sciences, University of Antwerp, 2160 Antwerp, Belgium; 160 Department of Medicine and Kovler Diabetes Center, University of Chicago, Chicago, IL USA; 161 School of Nursing, Faculty of Health Sciences, McMaster University, Hamilton, Canada; 162 Division of Endocrinology, Metabolism, Diabetes, University of Colorado, Boulder, CO USA; 163 Department of Clinical Medicine, School of Medicine, Trinity College Dublin, Dublin, Ireland; 164 Department of Endocrinology, Wexford General Hospital, Wexford, Ireland; 165 Division of Endocrinology, NorthShore University HealthSystem, Skokie, IL USA; 166 Department of Medicine, Prtizker School of Medicine, University of Chicago, Chicago, IL USA; 167 Department of Genetics, Stanford School of Medicine, Stanford University, Stanford, CA USA; 168 Faculty of Health, Aarhus University, Aarhus, Denmark; 169 Departments of Pediatrics and Medicine and Kovler Diabetes Center, University of Chicago, Chicago, USA; 170 Sanford Research, Sioux Falls, SD USA; 171 University of Washington School of Medicine, Seattle, WA USA; 172 Department of Population Medicine, Harvard Medical School, Harvard Pilgrim Health Care Institute, Boston, MA USA; 173 Department of Medicine, Universite de Sherbrooke, Sherbrooke, QC Canada; 174 Department of Internal Medicine, Seoul National University College of Medicine, Seoul National University Hospital, Seoul, Republic of Korea; 175 Joslin Diabetes Center, Harvard Medical School, Boston, MA USA; 176 Charles Bronfman Institute for Personalized Medicine, Icahn School of Medicine at Mount Sinai, New York, NY USA; 177 Broad Institute, Cambridge, MA USA; 178 Division of Metabolism, Digestion and Reproduction, Imperial College London, London, UK; 179 Department of Diabetes & Endocrinology, Imperial College Healthcare NHS Trust, London, UK; 180 Department of Diabetology, Madras Diabetes Research Foundation & Dr. Mohan’s Diabetes Specialities Centre, Chennai, India; 181 Department of Medicine, Faculty of Medicine and Health Sciences, University of Auckland, Auckland, New Zealand; 182 Auckland Diabetes Centre, Te Whatu Ora Health New Zealand, Auckland, New Zealand; 183 Medical Bariatric Service, Te Whatu Ora Counties, Health New Zealand, Auckland, New Zealand; 184 Oxford NIHR Biomedical Research Centre, University of Oxford, Oxford, UK; 185 University of Cambridge, Metabolic Research Laboratories and MRC Metabolic Diseases Unit, Wellcome-MRC Institute of Metabolic Science, Cambridge, UK; 186 Department of Epidemiology & Public Health, University of Maryland School of Medicine, Baltimore, MD USA; 187 Department of Internal Medicine, Division of Metabolism, Endocrinology and Diabetes, University of Michigan, Ann Arbor, MI USA; 188 AdventHealth Translational Research Institute, Orlando, FL USA; 189 Pennington Biomedical Research Center, Baton Rouge, LA USA; 190 Yale School of Medicine, New Haven, CT USA; 191 Faculty of Medicine and Health, University of Sydney, Sydney, NSW Australia; 192 Department of Endocrinology, Royal Prince Alfred Hospital, Sydney, NSW Australia; 193 Kaiser Permanente Northwest, Kaiser Permanente Center for Health Research, Portland, OR USA; 194 Clinial Research, Steno Diabetes Center Copenhagen, Herlev, Denmark; 195 Department of Clinical Medicine, Faculty of Health and Medical Sciences, University of Copenhagen, Copenhagen, Denmark; 196 Department of Endocrinology and Diabetology, University Hospital Düsseldorf, Heinrich Heine University Düsseldorf, Moorenstr. 5, 40225 Düsseldorf, Germany

**Keywords:** Diabetes, Endocrinology

Correction to: *Communications Medicine* 10.1038/s43856-023-00368-9, published online 05 October 2023

The file Supplementary Data 3 was missing when this article was published. It has now been added and can be accessed via the Supplementary information section in the online version of the article. Citations to Supplementary Data 3 in the article remain unchanged.

