# Supplementary material for: Genotype-stratified treatment for monogenic insulin resistance: a systematic review
Source: Commun Med (Lond). 2023 Oct 5;3:134. doi: 10.1038/s43856-023-00368-9 (PMC10550936; doi:10.1038/s43856-023-00368-9)
Supplement: Supplementary file 2 — Description of Additional Supplementary Data [file 43856_2023_368_MOESM2_ESM.pdf]

Description of Additional Supplementary Data

Genotype-stratified treatment for monogenic insulin resistance: a systematic review

Robert K. Semple, Kashyap A. Patel, Sungyoung Auh, ADA/EASD PMDI, Rebecca J. Brown

File Name: Supplementary Data 1

Description: Quality assessment of the 44 studies analysed, organized by affected gene and intervention.

File Name: Supplementary Data 2

Description: Subject-level outcome data extracted from the included studies, arranged by publication, affected gene, and intervention. Intervention duration, and pre- and post-treatment HbA1c, Body Mass Index (BMI), and triglycerides for all genes and treatments are in the first tab, “Main outcome measures.” Data for specific combinations of genes and treatments used to generate Figures 2, 3, and 4 are the remaining tabs.

File Name: Supplementary Data 3

Description: Summary statistics for intervention duration, and pre- and post-treatment and change in HbA1c, Body Mass Index (BMI), and triglycerides, and adverse events for combinations of genes and treatments.
