# Supplementary material for: Genotype-stratified treatment for monogenic insulin resistance: a systematic review
Source: Commun Med (Lond). 2023 Oct 5;3:134. doi: 10.1038/s43856-023-00368-9 (PMC10550936; doi:10.1038/s43856-023-00368-9)
Supplement: Supplementary file 6 — Supplementary Information [file 43856_2023_368_MOESM6_ESM.pdf]

Supplementary Information

Genotype-stratified treatment for monogenic insulin resistance: a systematic review

Robert K. Semple, Kashyap A. Patel, Sungyoung Auh, ADA/EASD PMDI, Rebecca J. Brown

Supplementary Table 1: Search Strategy for the Systematic Review

Disease	Gene Names and Variants	Treatments
Lipodystrophy	*LMNA*	Medication
Severe Insulin resistance	Lamin A/C	Therapy
Type A insulin resistance	*BSCL2*	Medical management
Donohue syndrome	Seipin	Treatment
Rabson Mendenhall syndrome	*AGPAT2*	SGLT2 inhibitor
Leprechaun	*PTRF*	SGLT2i
Leprechaunism	*CAVIN1*	SGLT-2 inhibitor
FPLD	*CAV1*	SGLT-2i
FPLD2	*ZMPSTE24*	Sodium Glucose Transporter 2 inhibitor
FPLD3	*PPARG*	Dapagliflozin
FPLD4	*PLIN1*	Empagliflozin
FPLD5	*MFN2*	Ertagliflozin
FPLD6	*CIDEC*	Canagliflozin
FPLD7	*LIPE*	Flozin
SHORT syndrome	*PCYT1A*	Thiazolidinedione
SOFT syndrome	*INSR*	PPARg Agonist
vSOFT	*AKT2*	PPAR gamma agonist
Werner syndrome	*PIK3R1*	TZD
Bloom syndrome	*AKT2*	Rosiglitazone
MOPDII	*WRN*	Pioglitazone
Osteodysplastic primordial dwarfism of Majewski type 2	*BLM*	Troglitazone
Alstrom syndrome	*NSMCE2*	GLP1RA
MANDIBULAR HYPOPLASIA, DEAFNESS, PROGEROID FEATURES, AND LIPODYSTROPHY SYNDROME	*POLD1*	GLP1 Receptor Agonists
MDPL	*PCNT*	GLP-1 Receptor Agonists
Mandibuloacral dysplasia	*POC1A*	GLP-1RA
MARFANOID-PROGEROID-LIPODYSTROPHY SYNDROME	*ALMS1*	Exenatide
MFLS	*PSMB8*	Liraglutide
PROTEASOME-ASSOCIATED AUTOINFLAMMATORY SYNDROME 1	*FBN1*	Lixisenatide
PRAAS1		Semaglutide

Robert K. Semple, Kashyap A. Patel, Sungyoung Auh, ADA/EASD PMDI, Rebecca J. Brown

		Dulaglutide
		Albiglutide
		Bariatric surgery
		Obesity Surgery
		Weight reduction surgery
		gastric band
		Roux-en-Y
		Gastric sleeve
		Gastric bypass
		metabolic surgery
		IGF-1
		rhIGF-1
		somatokine
		recombinant human insulin like growth factor 1
		Insulin like growth factor 1
		Increlex
		Mecasermin
		IGF-1/IGFBP3
		IGF1
		rhIGF1
		IGF1/IGFBP3
		leptin
		rhleptin
		metreleptin
		Myalept
		Myalepta

Legend: Search terms used to search PubMed, MEDLINE, and Embase included variations of the disease, gene, and treatment.

Supplementary Information

Genotype-stratified treatment for monogenic insulin resistance: a systematic review

Robert K. Semple, Kashyap A. Patel, Sungyoung Auh, ADA/EASD PMDI, Rebecca J. Brown

Supplementary Table 2: Number of individuals by intervention and genotype in included studies

		Treatment						
		rhIGF-1 or rhIGF-1/IGFBP3 composite	SGLT2i	TZD	Bariatric surgery	Metformin	Metreleptin	Total
**Gene**	*AGPAT2*	0	0	0	0	1	20	21
	BSCL2	0	1	1	0	0	19	21
	PTRF	0	0	0	0	0	1	1
	LMNA (progeroid)	0	0	0	0	0	2	2
	LMNA (FPL)	0	0	9	2	2	59	72
	PPARG	0	0	5	0	0	12	15
	PLIN1	0	0	0	2	0	0	2
	PIK3R1	0	1	0	0	0	0	1
	INSR	17	0	0	0	2	0	19
All partial LD		--	1	14	4	2	71	90
All generalised LD		--	1	1	0	1	40	43
All LD		--	2	15	4	3	111	135

Abbreviations: rhIGF-1, recombinant human insulin-like growth factor 1; IGFBP3, insulin-like growth factor binding protein 3; SGLT2i, sodium-glucose co-transporter-2 inhibitor; TZD, thiazolidinedione; FPL = Familial Partial Lipodystrophy

Supplementary Information

Genotype-stratified treatment for monogenic insulin resistance: a systematic review

Robert K. Semple, Kashyap A. Patel, Sungyoung Auh, ADA/EASD PMDI, Rebecca J. Brown

Supplementary Figure 1: Effects of Metreleptin therapy on glycated haemoglobin (A1c) by genotype

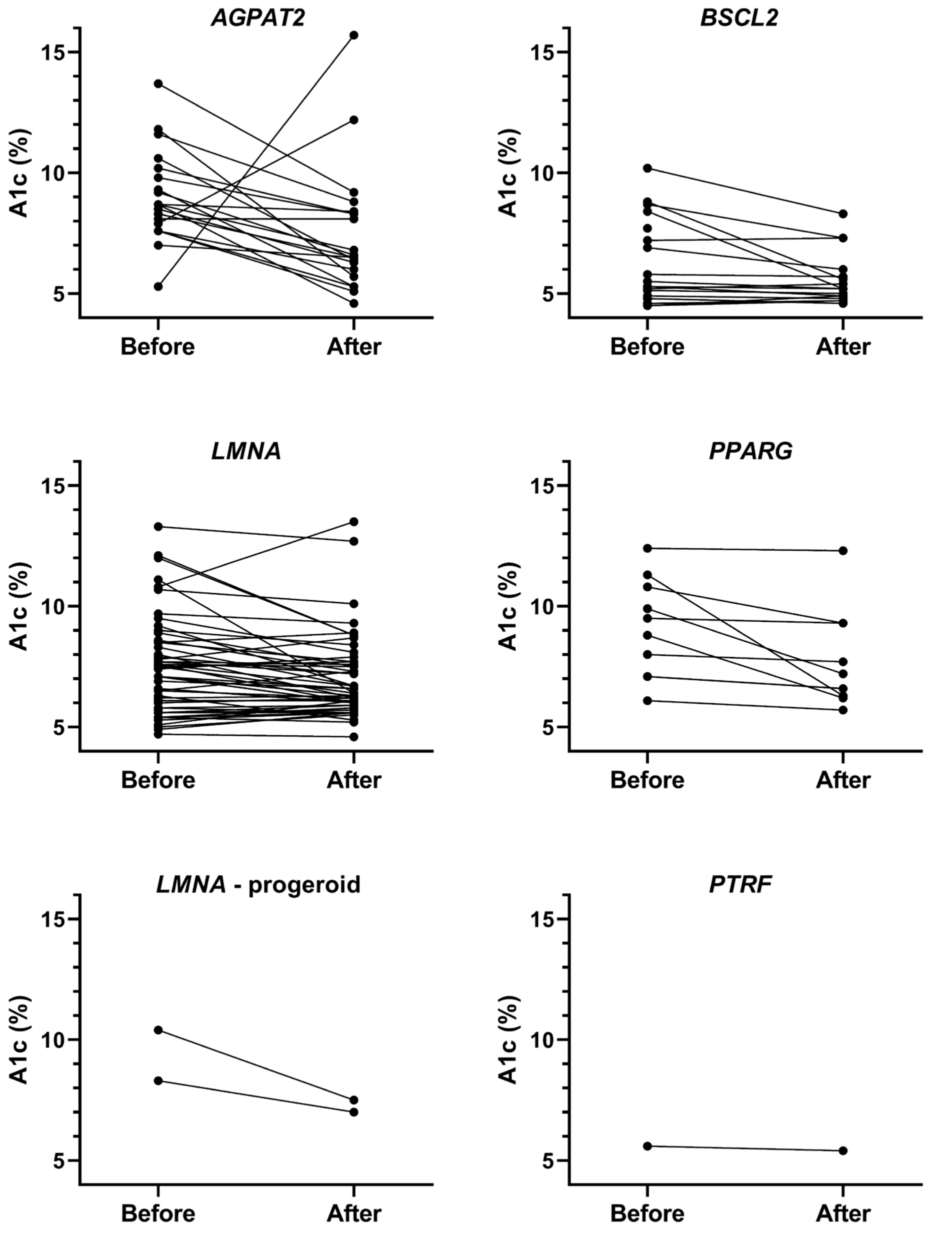

Supplementary Information

Genotype-stratified treatment for monogenic insulin resistance: a systematic review

Robert K. Semple, Kashyap A. Patel, Sungyoung Auh, ADA/EASD PMDI, Rebecca J. Brown

Supplementary Figure 2: Effects of Metreleptin therapy on serum triglyceride concentration by genotype

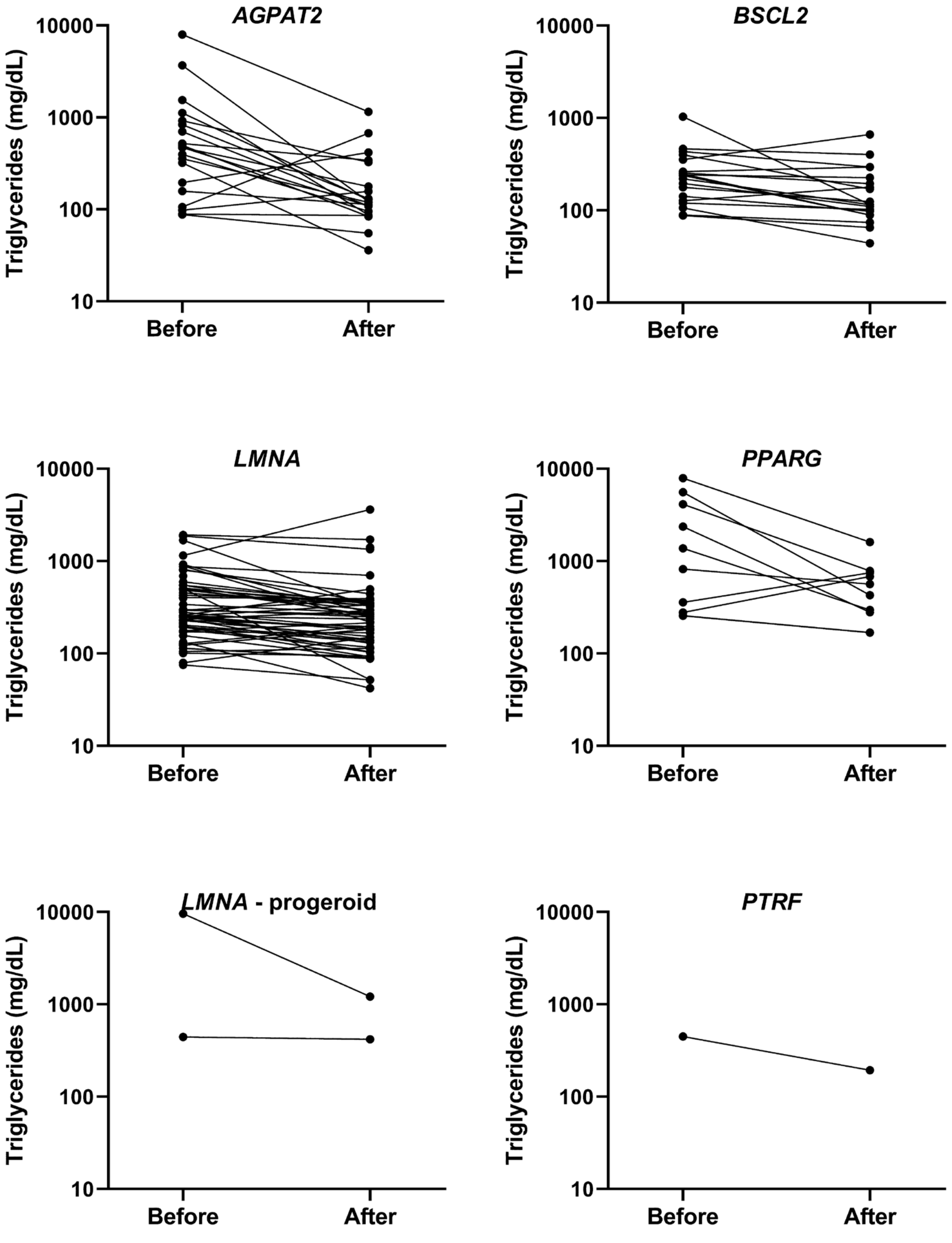

Supplementary Information

Genotype-stratified treatment for monogenic insulin resistance: a systematic review

Robert K. Semple, Kashyap A. Patel, Sungyoung Auh, ADA/EASD PMDI, Rebecca J. Brown

Supplementary Figure 3: Effects of Metreleptin therapy on body mass index (BMI) by genotype

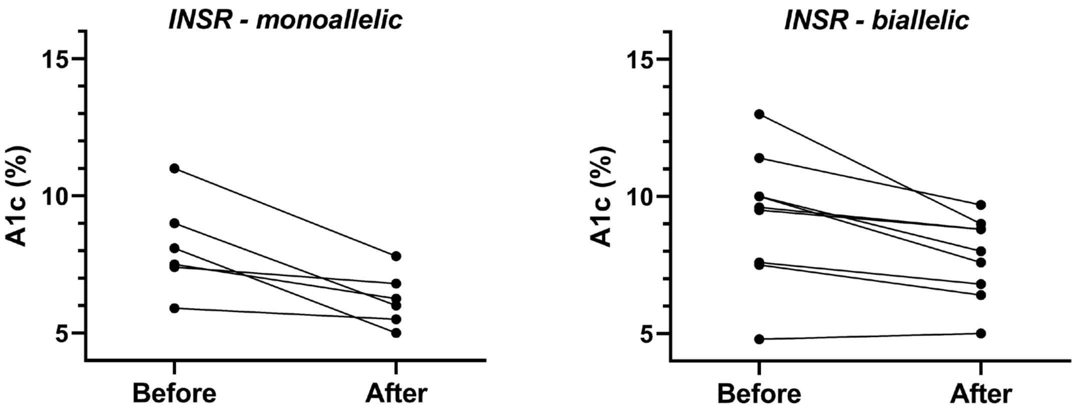

Supplementary Information

Genotype-stratified treatment for monogenic insulin resistance: a systematic review

Robert K. Semple, Kashyap A. Patel, Sungyoung Auh, ADA/EASD PMDI, Rebecca J. Brown

Supplementary Figure 4: Effects of recombinant IGF-1 or IGF-1 plus IGFBP3 therapy on glycated haemoglobin (A1c) in monallelic and biallelic insulin receptoropathy

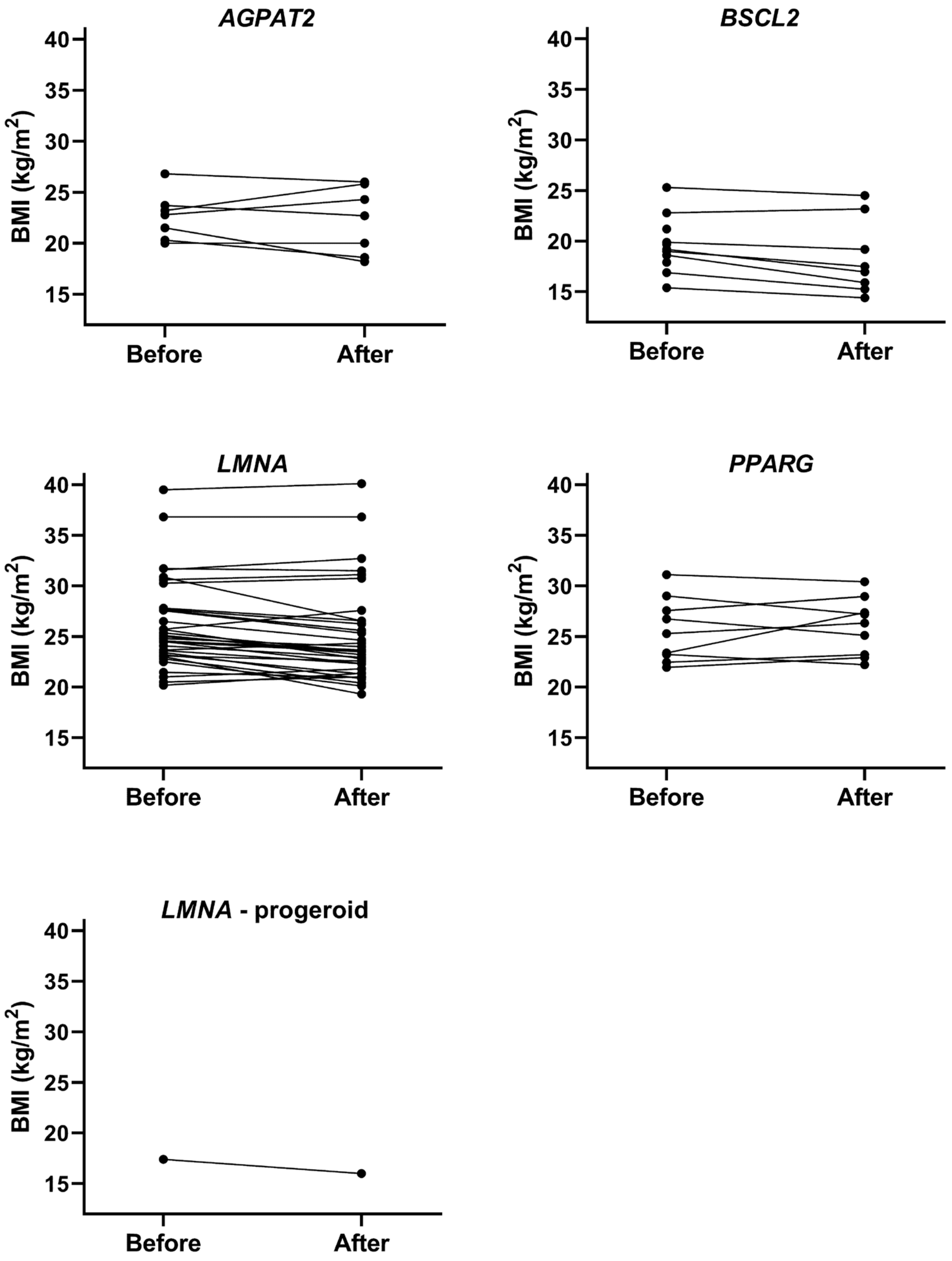

Genotype-stratified treatment for monogenic insulin resistance: a systematic review

Robert K. Semple, Kashyap A. Patel, Sungyoung Auh, ADA/EASD PMDI, Rebecca J. Brown

Supplementary Figure 5: Effects of thiazolidinedione therapy by genotype

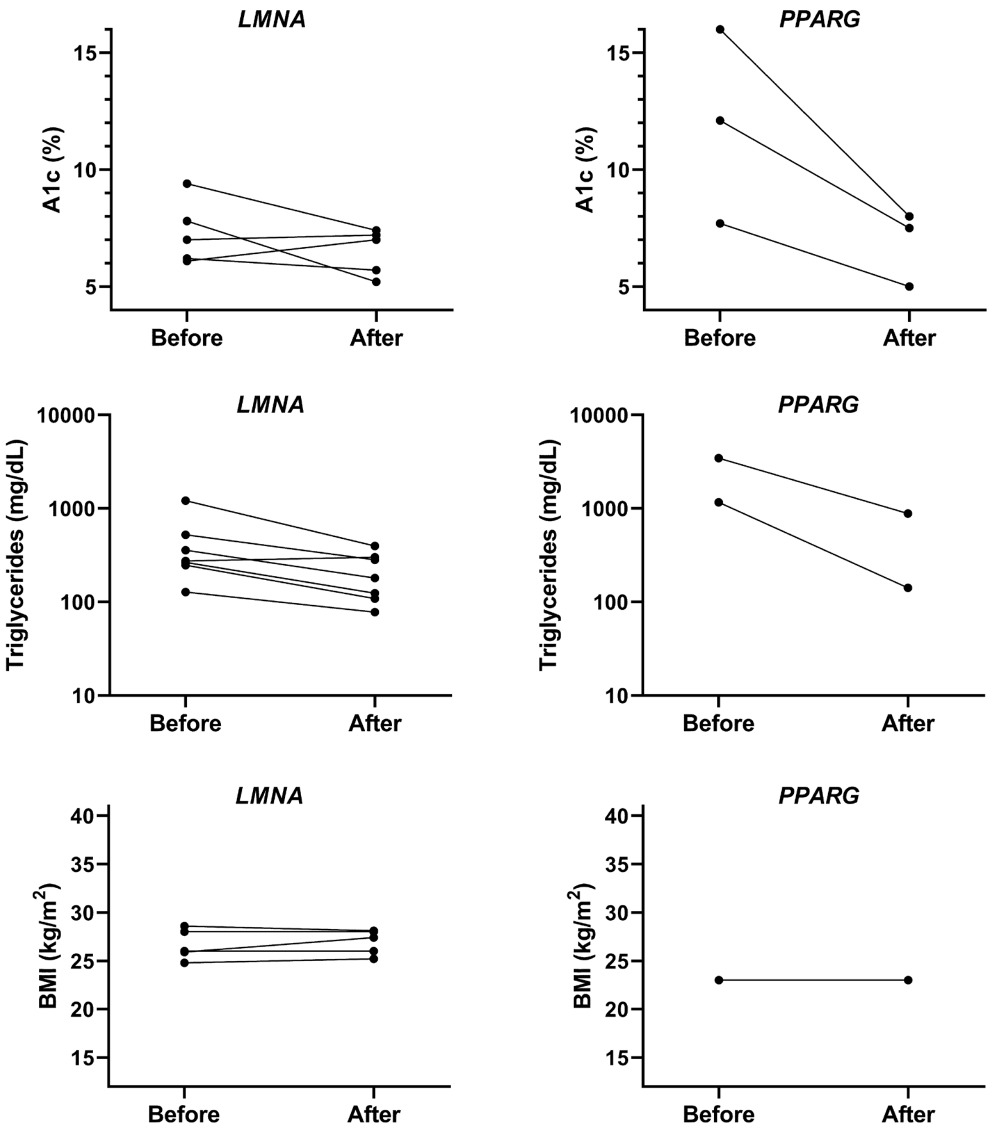

Genotype-stratified treatment for monogenic insulin resistance: a systematic review

Robert K. Semple, Kashyap A. Patel, Sungyoung Auh, ADA/EASD PMDI, Rebecca J. Brown

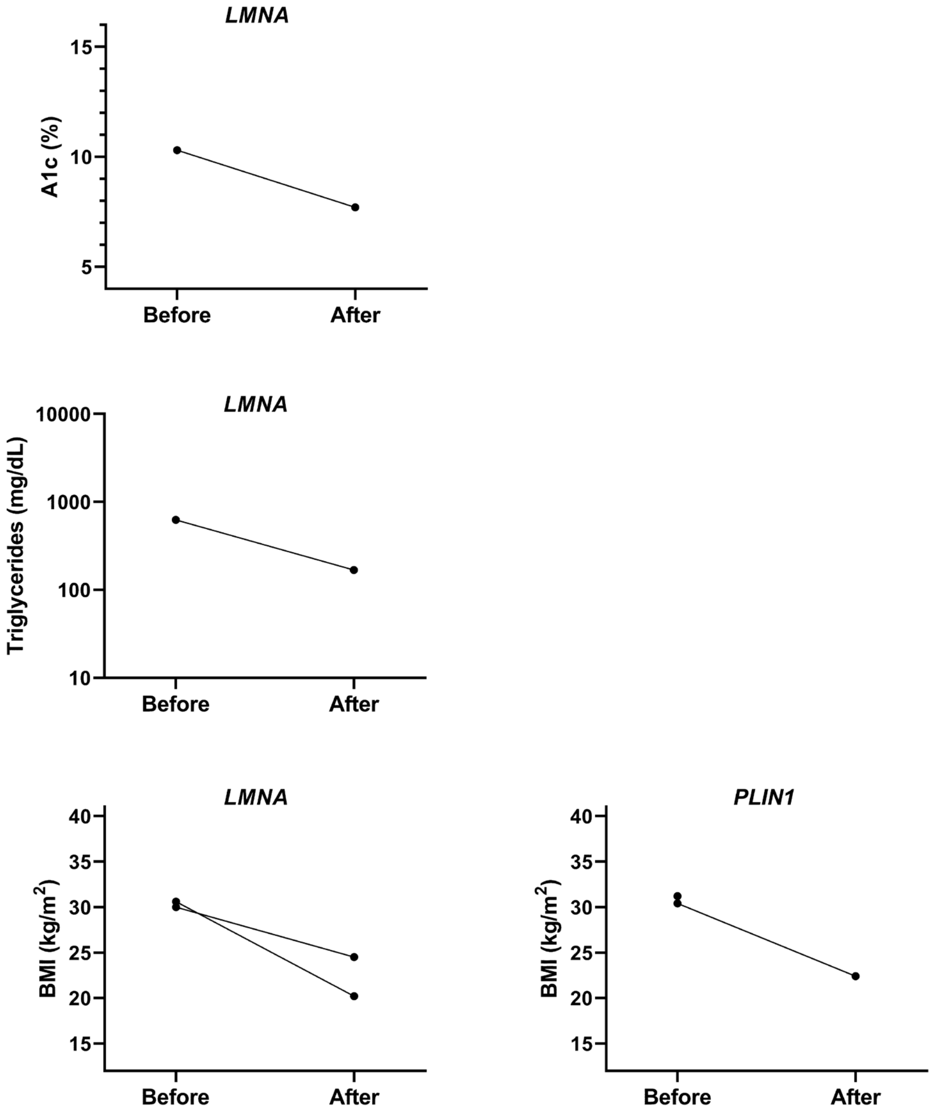

Supplementary Figure 6: Effects of bariatric surgery by genotype

Genotype-stratified treatment for monogenic insulin resistance: a systematic review

Robert K. Semple, Kashyap A. Patel, Sungyoung Auh, ADA/EASD PMDI, Rebecca J. Brown

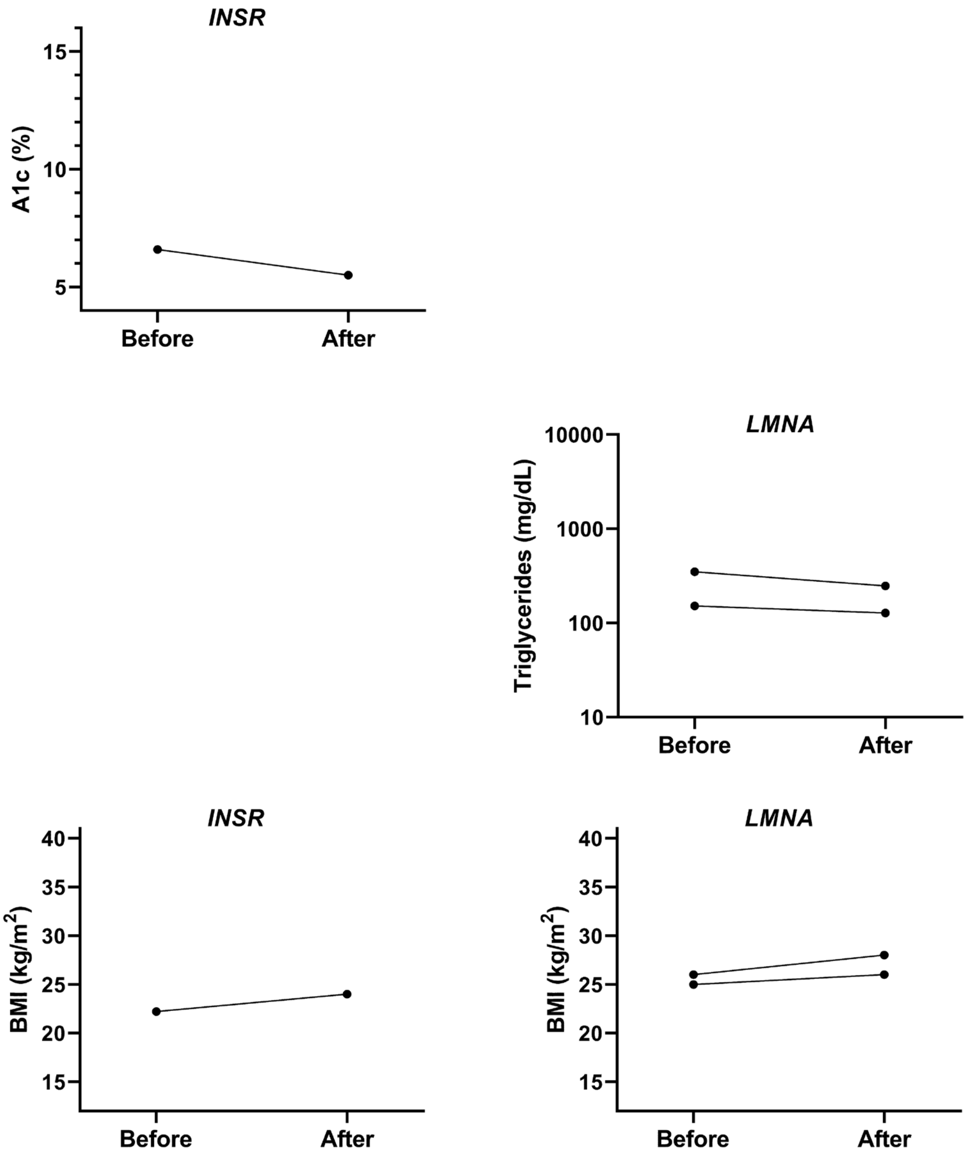

Supplementary Figure 7: Effects of metformin therapy by genotype

Genotype-stratified treatment for monogenic insulin resistance: a systematic review

Robert K. Semple, Kashyap A. Patel, Sungyoung Auh, ADA/EASD PMDI, Rebecca J. Brown

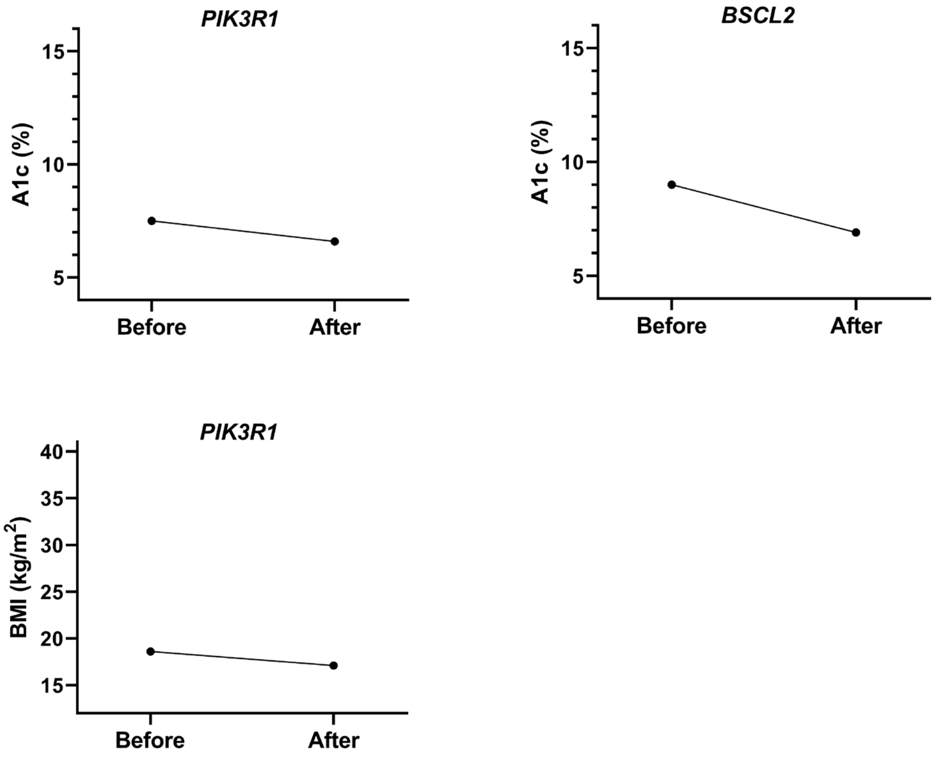

Supplementary Figure 8: Effects of SGLT2 inhibitor therapy by genotype
